# A study on the difference between functional connectivity in elbow flexion and extension according to object weight

**DOI:** 10.1097/MD.0000000000041781

**Published:** 2025-03-14

**Authors:** Jin-Seung Choi, Mi-Hyun Choi

**Affiliations:** a Biomedical Engineering, Research Institute of Biomedical Engineering, School of ICT Convergence Engineering, College of Science and Technology, Konkuk University, Chungju, South Korea.

**Keywords:** effect size, elbow flexion and extension, functional connectivity, object weight

## Abstract

In this study, the results of functional connectivity between various areas were extracted when flexion and extension of the elbow were repeatedly performed with 500 g and 1000 g balls in hand. All subjects performed elbow bending and stretching at an angle of 90 degrees with the right back of their hand facing down. A total of 30 bending and stretching exercises were performed for 1 minute. At this time, the experiment was carried out under 2 conditions: (500 g motion) when performing the exercise with a 500-g object in the right hand and a 1000 g object (1000 g motion). To process functional magnetic resonance imaging data, we used the Functional Connectivity (CONN) toolbox, which was implemented in Statistical Parametric Mapping software version 12. Visual.lateral (right, R)–Visual.medial, Visual.medical–Visual.occipital, SensoriMotor.superior–SensoriMotor.lateral (left, L), FrontoParital.lateral prefrontal cortex (R)–FrontoParital.posterior cingulate cortex (R), and DorsalAttention.intraparietal sulcus (R)–SensoriMotor.superior connection reduced the effect size when flexion and extension of the elbow joint with 1000 g objects more than 500 g objects were carried out. On the other hand, when the task was performed with 1000 g objects rather than 500 g, the effect size of Visual.lateral (L)–Visual.medial, Salience.supramarginal gyrus (L)–DorsalAttention.intraparietal sulcus (R), Language.posterior superior temporal gyrus (L)–Language.inferior frontal gyrus (L), and Salience.AInsula (L)–Salience.AInsula (R) connection increased. The connection that showed the highest correlation coefficient when performing elbow exercise with a 500 g object was DorsalAttention.frontal eye fields (L)–SensoriMotor.superior, Language.inferior frontal gyrus (L)–Language.inferior frontal gyrus (R), and DorsalAttention.intraparietal sulcus (R)–SensoriMotor.lateral. On the other hand, when performing elbow exercises with 1000 g of objects, the highest correlation coefficients were Language.posterior superior temporal gyrus (L)–Language.posterior superior temporal gyrus (R), and Salience.supramarginal gyrus (R)–DorsalAttention.intraparietal sulcus (R). In conclusion, these findings suggest that the brain adapts its functional connectivity patterns in response to changes in physical load during motor tasks, potentially implicating different cognitive and sensorimotor processes depending on the exertion level required by the task. This has implications for understanding the neural mechanisms underlying motor control and could inform rehabilitation strategies for motor impairments.

## 1. Introduction

The human brain is a remarkable and complex organ that orchestrates a myriad of processes, ranging from motor control to cognitive functions. Understanding how the brain’s functional connectivity (FC) adapts to different motor tasks and environmental conditions is pivotal in unraveling the intricacies of human neurophysiology.

Elbow flexion and extension exercises are fundamental movements that play a crucial role in various daily activities, from lifting objects to performing repetitive tasks. These actions inherently require the coordination of multiple brain regions responsible for motor control, sensory perception, and cognitive processing. Although the neural basis of these movements has been extensively studied,^[1–3]^ studies on the effect of manipulating the weight of objects maintained during these movements on brain FC are relatively insufficient.

Object weight is a variable that can significantly influence the neuromotor system response to a given task. The ability to adapt to different object weights reflects the brain’s remarkable plasticity and its capacity to optimize motor control strategies. Previous studies have demonstrated that manipulating object weight during motor tasks can lead to alterations in muscle activation patterns and kinematics.^[4]^ However, the neural mechanisms underlying these adaptations remain less understood.

Studies have shown that short-term exercise can enhance brain function and improve mental task performance. However, research on the impact of object weight changes during specific motor tasks on functional brain connectivity is relatively lacking. This study aims to analyze changes in functional brain connectivity when performing elbow flexion and extension tasks while handling objects of varying weights. Through this analysis, we seek to gain a deeper understanding of how the human brain adapts to environmental demands and motor tasks. Such understanding can provide valuable insights for designing rehabilitation programs and optimizing motor skill training. For instance, it can contribute to the development of tailored rehabilitation strategies for patients with neurological or musculoskeletal challenges who experience difficulties in performing motor tasks. The findings of this study can also be applied to the design of rehabilitation programs and the optimization of motor skill training. For example, adjusting the difficulty of motor tasks in the rehabilitation process of stroke patients may help facilitate functional brain recovery. Furthermore, in sports and physical therapy, training methods that consider brain network activation related to specific object weights can help improve motor performance.

In this article, we present the results of our investigation into the FC patterns of the human brain during elbow flexion and extension exercises while holding objects of varying weights: a 500-g and a 1000-g ball. We employed functional magnetic resonance imaging (fMRI) to capture the dynamic changes in brain FC associated with these motor tasks. Our primary aim was to identify specific FC networks that respond differentially to the manipulation of object weight, shedding light on the intricate interplay between motor control and cognitive processing.

## 2. Methods

An experiment was conducted on 10 healthy male college students (22.4 ± 1.2 years old) in their 20s who had no experience of brain damage and whose cognitive processing was normal. In addition, healthy men without osteoarthritis capable of performing natural movements in movement were selected. People with metal substances such as pacemakers and iron cores that can affect magnetic resonance (MR) images during MR imaging were limited in the scope of recruitment. External factors such as smoking, alcohol, and coffee that can affect brain activation were limited to subjects before the experiment and the purpose and contents of the experiment were fully explained. This study was approved by the Institutional Review Board of Konkuk University (IRB project number: 7001355-202103-HR-426) and complied with the regulations of the Helsinki Declaration. Before the experiment, participants were informed about the study and provided written informed consent.

Since this experiment required participants to perform arm movements inside an MR scanner, steps were taken to minimize movement during MR image acquisition. Before the experiment, participants practiced flexion and extension of the elbow at a 90-degree angle to familiarize themselves with the task. During the experiment, the researcher continuously monitored real-time MR images to ensure that participants’ movements were not excessive. If excessive movement was detected, the experiment was immediately paused, the participant’s posture was readjusted, and the experiment was restarted.

Each block consists of a rest (1 minute) section and a lift (1 minute) section, and the total experiment time was 8 minutes. In the lift section, all subjects performed elbow bending and stretching at an angle of 90 degrees with the right back of their hand facing down. A total of 30 bending and stretching exercises were performed for 1 minute. At this time, the experiment was carried out under 2 conditions: (500 g motion) when performing the exercise with a 500-g object in the right hand and a 1000-g object (1000 g motion). The experiment was performed randomly, and after the experiment under any condition (500 g motion or 1000 g motion) was completed, a 20-minute break was taken and another condition was performed. Before performing the experiment, all subjects practiced the bending and stretching of the right elbow at an angle of 90 degrees and then performed the experiment. A plastic ball that did not affect the MR image was used for objects of 500 g and 1000 g.

For the acquisition of brain function images, 3T ISOL Technology FORTE at the KAIST Brain Science Research Center was used, and 35 brain section images per volume were obtained by the single-shot echo planar imaging method (repetition time/echo time: 3000/35 ms, field of view: 240 mm, matrix: 64 × 64, slice thickness: 4 mm). Anatomical brain images were obtained by the T1-weighted imaging method (repetition time/echo time: 280/14 ms, field of view: 240 mm, matrix: 256 × 256, slice thickness: 4 mm).

The fMRI data analysis was performed using SPM12 (Statistical Parametric Mapping 12; Wellcome Department of Cognitive Neurology, London, UK) and the CONN toolbox (Functional Connectivity Toolbox, https://web.conn-toolbox.org/). The preprocessing and analysis procedures followed the methodology established in a previous study,^[5]^ and the key steps are as follows.

Functional images were preprocessed using SPM12, including realignment, coregistration, normalization (using the MNI template), and 8-mm Gaussian smoothing. Motion correction was performed using a 6-parameter rigid body transformation, and a 240-second high-pass filter was applied to remove high-frequency noise.

FC analysis was conducted using the CONN toolbox based on region of interest (ROI)-to-ROI analysis. A total of 105 ROIs were defined according to the Harvard–Oxford cortical and subcortical structural atlases (https://neurovault.org/collections/262/), excluding the brainstem and cerebellum. We selected statistically significant ROI-to-ROI connections that correlate with the performance of the recognition tests by cluster-level inferences. The *T*-statistics of the entire ROI-to-ROI matrix were estimated using a general linear model.

Seeds networks selected 7 networks and 19 ROIs, including the medial prefrontal cortex, lateral parietal lobe (LP), and posterior cingulate cortex (PCC) regions of the Default Mode network; lateral and superior regions of the SensoriMotor network; and medial, occipital, and lateral areas of the visual network were selected. In addition, the anterior cingulate cortex, anterior insular cortex, rostral prefrontal cortex, and supramarginal gyrus (SMG) of the salience network, frontal eye fields (FEF), intraparietal sulcus (IPS), and frontal gyrus of the dorsal attention network (DAN), and the lateral prefrontal cortex (LPFC) and posterior parietal cortex (PPC) of the frontoparietal network (FPN) were selected. In the language network, inferior frontal gyrus (IFG) and posterior superior temporal gyrus (pSTG) regions were selected for analysis.

Thereafter, this statistical parametric map was thresholded using a significance level of *P* < .05 (uncorrected). The resulting suprathreshold areas define a series of nonoverlapping clusters. The cluster-level false discovery rate-corrected *P* < .05 was applied.

## 3. Results

**Figure 1.**
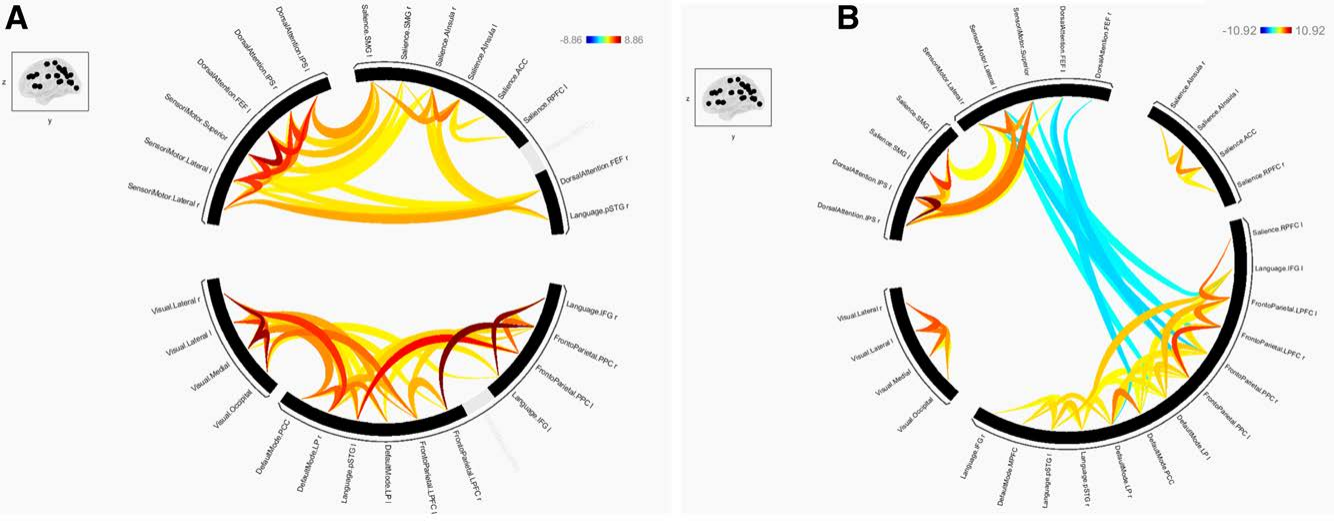
The connection of each network for the 2 conditions: (A) 500 g motion and (B) 1000 g motion.

**Figure 2.**
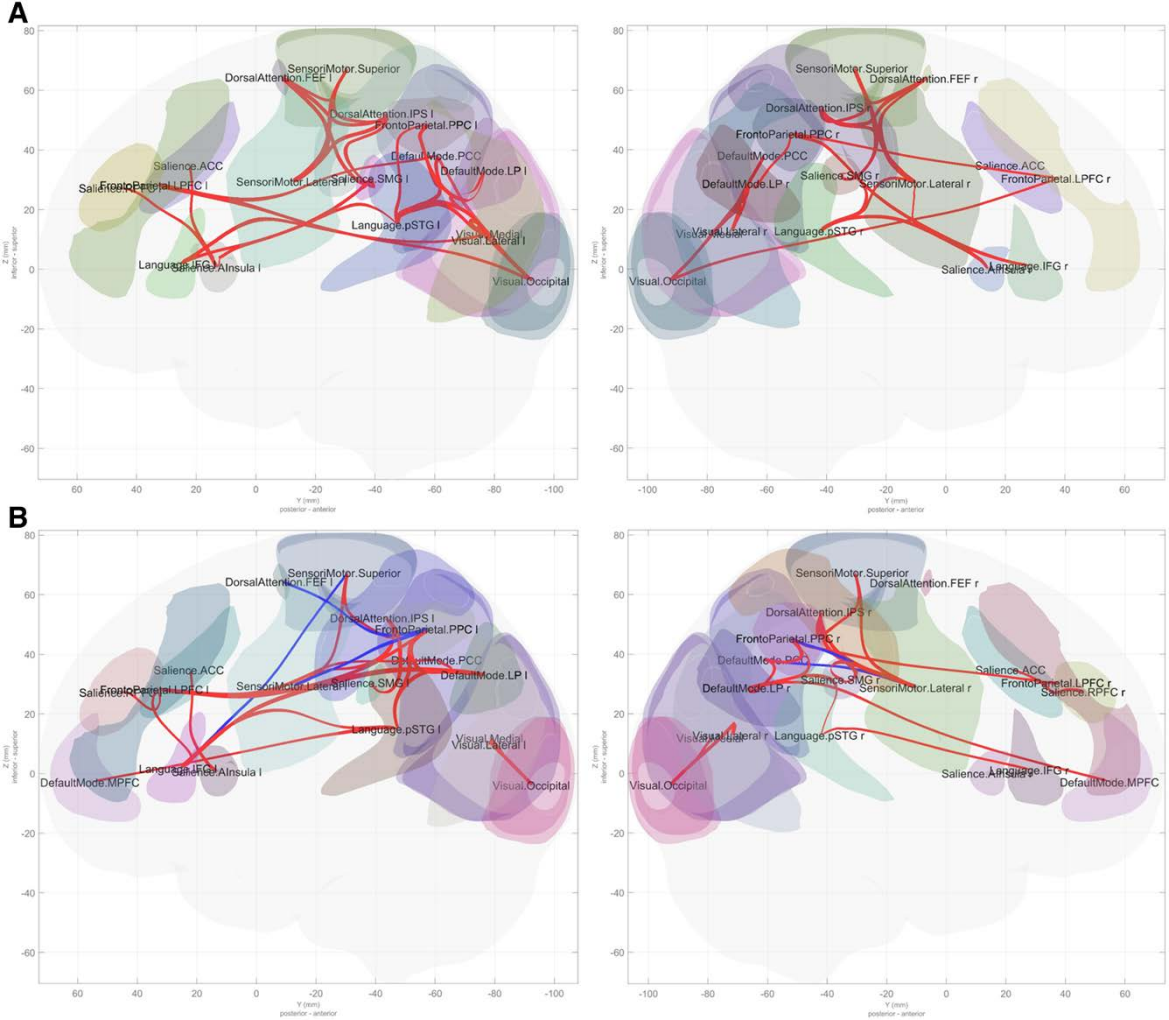
The connection of each network for the 2 conditions: (A) 500 g motion and (B) 1000 g motion.

The results of FC during flexion and extension of elbow joints under both conditions (500 g motion and 1000 g motion) are shown in Tables 1 and 2, and the connection of each network for the 2 conditions is presented in Figure 1 in the form of a ring display, and in Figure 2 in the form of a glass display, respectively.

**Table 1:** Functional connectivity results derived from performing elbow flexion and extension with 500 g object (connection network and effect size).

Analysis unit	Statistic	
	*t* value	Effect size
Cluster 1/10	*F*(1,8) = 77.04	
DorsalAttention.FEF (L) (−27, −9, 64)–SensoriMotor.superior (0, −31, 67)	7.96	0.51
SensoriMotor.lateral (L) (−55, −12, 29)–SensoriMotor.lateral (R) (56, −10, 29)	5.69	0.82
SensoriMotor.Superior (0, −31, 67)–SensoriMotor.lateral (L) (−55, −12, 29)	6.03	0.48
DorsalAttention.IPS (L) (−39, −43, 52)–DorsalAttention.FEF (L) (−27, −9, 64)	6.33	0.33
DorsalAttention.IPS (R) (39, −42, 54)–SensoriMotor.lateral (L) (−55, −12, 29)	5.86	0.43
DorsalAttention.IPS (R) (39, −42, 54)–SensoriMotor.superior (0, −31, 67)	5.36	0.49
DorsalAttention.IPS (L) (−39, −43, 52)–DorsalAttention.IPS (R) (39, −42, 54)	5.61	0.67
DorsalAttention.FEF (L) (−27, −9, 64)–SensoriMotor.lateral (L) (−55, −12, 29)	4.55	0.29
DorsalAttention.IPS (L) (−39, −43, 52)–SensoriMotor.superior (0, −31, 67)	4.52	0.36
DorsalAttention.IPS (L) (−39, −43, 52)–SensoriMotor.lateral (L) (−55, −12, 29)	4.33	0.49
SensoriMotor.superior (0, −31, 67)–SensoriMotor.lateral (R) (56, −10, 29)	2.86	0.33
DorsalAttention.IPS (R) (39, −42, 54)–SensoriMotor.lateral (R) (56, −10, 29)	2.86	0.30
Cluster 2/10	*F*(1,8) = 42.15	
Visual.medial (2, −79, 12)–Visual.occipital (0, −93, −4)	8.82	0.44
Visual.lateral (R) (38, −72, 13)–Visual.medial (2, −79, 12)	8.52	0.67
Visual.lateral (L) (−37, −79, 10)–Visual.occipital (0, −93, −4)	5.76	0.51
Visual.lateral (R) (38, −72, 13)–Visual.occipital (0, −93, −4)	4.43	0.39
Visual.lateral (R) (38, −72, 13)–Visual.lateral (L) (−37, −79, 10)	3.47	0.52
Visual.lateral (L) (−37, −79, 10)–Visual.medial (2, −79, 12)	3.06	0.43
Cluster 3/10	*F*(1,8) = 25.53	
FrontoParietal.LPFC (R) (41, 38, 30)–FrontoParietal.PPC (R) (52, −52, 45)	8.86	0.34
Language.IFG (L) (−51, 26, 2)–Language.IFG (R) (54, 28, 1)	8.62	0.48
Language.pSTG (L) (−57, −47, 15)–FrontoParietal.PPC (L) (−46, −58, 49)	6.68	0.24
DefaultMode.LP (R) (47, −67, 29)–DefaultMode.LP (L) (−39, −77, 33)	4.49	0.32
FrontoParietal.PPC (L) (−46, −58, 49)–FrontoParietal.PPC (R) (52, −52, 45)	4.87	0.44
DefaultMode.PCC (1, −61, 38)–DefaultMode.LP (R) (47, −67, 29)	5.1	0.36
DefaultMode.PCC (1, −61, 38)–Language.pSTG (L) (−57, −47, 15)	4.2	0.21
DefaultMode.LP (R) (47, −67, 29)–FrontoParietal.LPFC (L) (−43, 33, 28)	3.64	0.30
FrontoParietal.LPFC (L) (−43, 33, 28)–FrontoParietal.LPFC (R) (41, 38, 30)	3.52	0.33
DefaultMode.LP (R) (47, −67, 29)–FrontoParietal.PPC (R) (52, −52, 45)	3.02	0.29
DefaultMode.PCC (1, −61, 38)–FrontoParietal.LPFC (L) (−43, 33, 28)	3.41	0.18
FrontoParietal.LPFC (L) (−43, 33, 28)–FrontoParietal.PPC (R) (52, −52, 45)	2.91	0.23
FrontoParietal.PPC (L) (−46, −58, 49)–Language.IFG (R) (54, 28, 1)	3.51	0.34
DefaultMode.LP (R) (47, −67, 29)–Language.pSTG (L) (−57, −47, 15)	2.61	0.23
DefaultMode.LP (R) (47, −67, 29)–Language.IFG (L) (−51, 26, 2)	2.45	0.27
FrontoParietal.PPC (R) (52, −52, 45)–Language.IFG (R) (54, 28, 1)	3.02	0.17
Language.pSTG (L) (−57, −47, 15)–Language.IFG (L) (−51, 26, 2)	2.64	0.17
DefaultMode.PCC (1, −61, 38)–FrontoParietal.PPC (L) (−46, −58, 49)	2.59	0.14
DefaultMode.LP (L) (−39, −77, 33)–FrontoParietal.PPC (L) (−46, −58, 49)	2.43	0.25
Cluster 4/10	*F*(1,8) = 18.00	
Visual.lateral (R) (38, −72, 13)–DefaultMode.LP (R) (47, −67, 29)	5.86	0.33
Visual.occipital (0, −93, −4)–FrontoParietal.LPFC (L) (−43, 33, 28)	4.75	0.30
Visual.medial (2, −79, 12)–DefaultMode.PCC (1, −61, 38)	4.17	0.33
Visual.lateral (R) (38, −72, 13)–FrontoParietal.LPFC (L) (−43, 33, 28)	3.74	0.26
Visual.medial (2, −79, 12)–DefaultMode.LP (R) (47, −67, 29)	3.11	0.32
Visual.occipital (0, −93, −4)–FrontoParietal.LPFC (R) (41, 38, 30)	2.5	0.16
Visual.lateral (L) (−37, −79, 10)–DefaultMode.LP (R) (47, −67, 29)	2.74	0.20
Visual.lateral (L) (−37, −79, 10)–Language.pSTG (L) (−57, −47, 15)	2.65	0.25
Visual.lateral (R) (38, −72, 13)–Language.pSTG (L) (−57, −47, 15)	2.38	0.17
Visual.medial (2, −79, 12)–Language.IFG (L) (−51, 26, 2)	2.42	0.15
Visual.medial (2, −79, 12)–Language.pSTG (L) (−57, −47, 15)	2.33	0.18
Visual.lateral (L) (−37, −79, 10)–DefaultMode.LP (L) (−39, −77, 33)	2.36	0.23
Cluster 5/10	*F*(1,8) = 12.29	
Salience.SMG (L) (−60, −39, 31)–DorsalAttention.IPS (L) (−39, −43, 52)	3.88	0.46
Language.pSTG (R) (59, −42, 13)–SensoriMotor.lateral (R) (56, −10, 29)	3.71	0.30
Salience.SMG (L) (−60, −39, 31)–DorsalAttention.FEF (L) (−27, −9, 64)	3.23	0.22
Salience.SMG (L) (−60, −39, 31)–DorsalAttention.IPS (R) (39, −42, 54)	2.79	0.18
Salience.AInsula (R) (47, 14, 0)–SensoriMotor.lateral (R) (56, −10, 29)	2.41	0.22
Salience.AInsula (R) (47, 14, 0)–SensoriMotor.lateral (L) (−55, −12, 29)	2.33	0.20
Salience.SMG (R) (62, −35, 32)–SensoriMotor.superior (0, −31, 67)	2.79	0.24
Salience.SMG (R) (62, −35, 32)–SensoriMotor.lateral (L) (−55, −12, 29)	2.42	0.24
DorsalAttention.FEF (R) (30, −6, 64)–SensoriMotor.lateral (R) (56, −10, 29)	3.36	0.27
DorsalAttention.FEF (R) (30, −6, 64)–SensoriMotor.lateral (L) (−55, −12, 29)	2.65	0.23
DorsalAttention.FEF (R) (30, −6, 64)–SensoriMotor.superior (0, −31, 67)	2.61	0.28
DorsalAttention.FEF (R) (30, −6, 64)–DorsalAttention.IPS (R) (39, −42, 54)	2.45	0.25
Cluster 6/10	*F*(1,8) = 7.49	
Salience.AInsula (L) (−44, 13, 1)–Salience.AInsula (R) (47, 14, 0)	4.55	0.35
Salience.AInsula (L) (−44, 13, 1)–Salience.SMG (L) (−60, −39, 31)	3.88	0.21
Salience.AInsula (R) (47, 14, 0)–Salience.SMG (L) (−60, −39, 31)	3.67	0.30
Language.pSTG (R) (59, −42, 13)–Salience.AInsula (L) (−44, 13, 1)	2.86	0.12
Language.pSTG (R) (59, −42, 13)–Salience.SMG (R) (62, −35, 32)	2.73	0.21
Language.pSTG (R) (59, −42, 13)–Salience.AInsula (R) (47, 14, 0)	2.53	0.20
Salience.ACC (0, 22, 35) –Salience.AInsula (L) (−44, 13, 1)	2.56	0.29
Salience.RPFC (L) (−32, 45, 27)–Salience.AInsula (L) (−44, 13, 1)	2.54	0.17

ACC = anterior cingulate cortex, Alnsula = anterior insular cortex, FEF = frontal eye fields, IFG = inferior frontal gyrus, IPS = intraparietal sulcus, LP = lateral parietal lobe, LPFC = lateral prefrontal cortex, PCC = posterior cingulate cortex, PPC = posterior cingulate cortex, pSTG = posterior superior temporal gyrus, RPFC = rostral prefrontal cortex, SMG = supramarginal gyrus.

**Table 2:** Functional connectivity results derived from performing elbow flexion and extension with 1000 g object (connection network and effect size).

Analysis unit	Statistic	
	*t* value	Effect size
Cluster 1/15	*F*(1,8) = 169.93	
Visual.lateral (R) (38, −72, 13)–Visual.lateral (L) −37, −79, 10)	6.6	0.70
Visual.lateral (R) (38, −72, 13)–Visual.medial (2, −79, 12)	5.89	0.57
Visual.lateral (L) (−37, −79, 10)–Visual.medial (2, −79, 12)	5.6	0.58
Visual.lateral (R) (38, −72, 13)–Visual.occipital (0, −93, −4)	4.64	0.31
Visual.lateral (L) (−37, −79, 10)–Visual.occipital (0, −93, −4)	4.57	0.46
Visual.medial (2, −79, 12)–Visual.occipital (0, −93, −4)	2.24	0.25
Cluster 2/15	*F*(1,8) = 65.60	
DorsalAttention.IPS (L) (−39, −43, 52)–DorsalAttention.IPS (R) (39, −42, 54)	10.92	0.82
Salience.SMG (R) (62, −35, 32)–Salience.SMG (L) (−60, −39, 31)	7.08	0.34
Salience.SMG (L) (−60, −39, 31)–DorsalAttention.IPS (L) (−39, −43, 52)	4.64	0.47
Salience.SMG (L) (−60, −39, 31)–DorsalAttention.IPS (R) (39, −42, 54)	3.5	0.31
Salience.SMG (R) (62, −35, 32)–DorsalAttention.IPS (R) (39, −42, 54)	3.38	0.37
Salience.SMG (R) (62, −35, 32)–DorsalAttention.IPS (L) (−39, −43, 52)	3.1	0.27
Cluster 3/15	*F*(1,8) = 37.27	
SensoriMotor.lateral (L) (−55, −12, 29)–SensoriMotor.lateral (R) (56, −10, 29)	5.73	0.73
SensoriMotor.superior (0, −31, 67)–SensoriMotor.lateral (R) (56, −10, 29)	4.57	0.26
SensoriMotor.superior (0, −31, 67)–SensoriMotor.lateral (L) (−55, −12, 29)	4.22	0.36
Cluster 4/15	*F*(1,8) = 33.34	
FrontoParietal.PPC (L) (−46, −58, 49)–FrontoParietal.LPFC (R) (41, 38, 30)	7.03	0.33
FrontoParietal.LPFC (L) (−43, 33, 28)–Salience.RPFC (L) (−32, 45, 27)	5.87	0.30
FrontoParietal.LPFC (R) (41, 38, 30)–FrontoParietal.LPFC (L) (−43, 33, 28)	5.48	0.44
DefaultMode.LP (R) (47, −67, 29)–DefaultMode.PCC (1, −61, 38)	5.5	0.38
DefaultMode.LP (R) (47, −67, 29)–FrontoParietal.PPC (L) (−46, −58, 49)	3.32	0.30
DefaultMode.LP (R) (47, −67, 29)–DefaultMode.LP (L) (−39, −77, 33)	3.23	0.34
DefaultMode.LP (R) (47, −67, 29)–FrontoParietal.PPC (R) (52, −52, 45)	3.07	0.30
FrontoParietal.PPC (L) (−46, −58, 49)–FrontoParietal.PPC (R) (52, −52, 45)	3.84	0.37
FrontoParietal.LPFC (L) (−43, 33, 28)–Language.IFG (L) (−51, 26, 2)	3.59	0.22
Language.pSTG (L) (−57, −47, 15)–Language.IFG (L) (−51, 26, 2)	3.86	0.23
Language.pSTG (L) (−57, −47, 15)–Language.pSTG (R) (59, −42, 13)	3.73	0.42
FrontoParietal.PPC (R) (52, −52, 45)–FrontoParietal.LPFC (R) (41, 38, 30)	3.43	0.28
DefaultMode.LP (L) (−39, −77, 33)–Language.IFG (L) (−51, 26, 2)	4.1	0.13
DefaultMode.LP (L) (−39, −77, 33)–FrontoParietal.PPC (L) (−46, −58, 49)	3.69	0.33
FrontoParietal.PPC (L) (−46, −58, 49)–FrontoParietal.LPFC (L) (−43, 33, 28)	2.75	0.24
Language.pSTG (L) (−57, −47, 15)–FrontoParietal.PPC (L) (−46, −58, 49)	2.79	0.27
Language.pSTG (R) (59, −42, 13)–DefaultMode.LP (R) (47, −67, 29)	3.12	0.29
Language.pSTG (L) (−57, −47, 15)–DefaultMode.LP (L) (−39, −77, 33)	2.66	0.23
DefaultMode.MPFC (1, 55, −3)–DefaultMode.LP (R) (47, −67, 29)	3.52	0.22
DefaultMode.MPFC (1, 55, −3)–Language.pSTG (L) (−57, −47, 15)	3.3	0.31
DefaultMode.PCC (1, −61, 38)–FrontoParietal.PPC (R) (52, −52, 45)	3.23	0.21
DefaultMode.PCC (1, −61, 38)–FrontoParietal.LPFC (L) (−43, 33, 28)	2.98	0.27
DefaultMode.LP (L) (−39, −77, 33)–FrontoParietal.LPFC (L) (−43, 33, 28)	2.5	0.25
DefaultMode.PCC (1, −61, 38)–DefaultMode.LP (L) (−39, −77, 33)	2.45	0.24
FrontoParietal.PPC (R) (52, −52, 45)–Language.IFG (L) (−51, 26, 2)	2.43	0.25
Language.IFG (R) (54, 28, 1)–Language.pSTG (L) (−57, −47, 15)	3.09	0.24
Language.IFG (R) (54, 28, 1)–Language.pSTG (R) (59, −42, 13)	2.52	0.23
Cluster 5/15	*F*(1,8) = 13.93	
SensoriMotor.superior (0, −31, 67)–DorsalAttention.IPS (R) (39, −42, 54)	5.75	0.33
SensoriMotor.superior (0, −31, 67)–DorsalAttention.IPS (L) (−39, −43, 52)	5.72	0.37
SensoriMotor.lateral (L) (−55, −12, 29)–DorsalAttention.IPS (L) (−39, −43, 52)	5.03	0.43
SensoriMotor.lateral (R) (56, −10, 29)–Salience.SMG (R) (62, −35, 32)	2.67	0.27
SensoriMotor.lateral (R) (56, −10, 29)–DorsalAttention.IPS (R) (39, −42, 54)	2.53	0.27
DorsalAttention.FEF (L) (−27, −9, 64)–DorsalAttention.IPS (R) (39, −42, 54)	2.52	0.24
Cluster 6/15	*F*(1,8) = 13.22	
DefaultMode.LP (R) (47, −67, 29)–DorsalAttention.FEF (L) (−27, −9, 64)	−3.37	−0.15
FrontoParietal.PPC (L) (−46, −58, 49)–DorsalAttention.FEF (L) (−27, −9, 64)	−3.54	−0.22
FrontoParietal.PPC (R) (52, −52, 45)–SensoriMotor.lateral (R) (56, −10, 29)	−3.49	−0.18
FrontoParietal.PPC (L) (−46, −58, 49)–DorsalAttention.FEF (R) (30, −6, 64)	−2.89	−0.11
FrontoParietal.PPC (L) (−46, −58, 49)–SensoriMotor.lateral (L) (−55, −12, 29)	−2.65	−0.23
FrontoParietal.LPFC (R) (41, 38, 30)–DorsalAttention.FEF (L) (−27, −9, 64)	−2.89	−0.09
FrontoParietal.LPFC (R) (41, 38, 30)–SensoriMotor.lateral (L) (−55, −12, 29)	−2.73	−0.17
DefaultMode.PCC (1, −61, 38)–SensoriMotor.lateral (R) (56, −10, 29)	−2.58	−0.14
Language.IFG (L) (−51, 26, 2)–SensoriMotor.superior (0, −31, 67)	−2.47	−0.17
Cluster 7/15	*F*(1,8) = 12.34	
Salience.ACC (0, 22, 35) –Salience.AInsula (L) (−44, 13, 1)	5.23	0.30
Salience.RPFC (R) (32, 46, 27)–Salience.AInsula (L) (−44, 13, 1)	3.07	0.23
Salience.RPFC (R) (32, 46, 27)–Salience.ACC (0, 22, 35)	3.01	0.27
Salience.AInsula (L) (−44, 13, 1)–Salience.AInsula (R) (47, 14, 0)	3.62	0.44

ACC = anterior cingulate cortex, Alnsula = anterior insular cortex, FEF = frontal eye fields, IFG = inferior frontal gyrus, IPS = intraparietal sulcus, LP = lateral parietal lobe, LPFC = lateral prefrontal cortex, MPFC = medial prefrontal cortex, PCC = posterior cingulate cortex, PPC = posterior cingulate cortex, pSTG = posterior superior temporal gyrus, RPFC = rostral prefrontal cortex, SMG = supramarginal gyrus.

**Figure 3.**
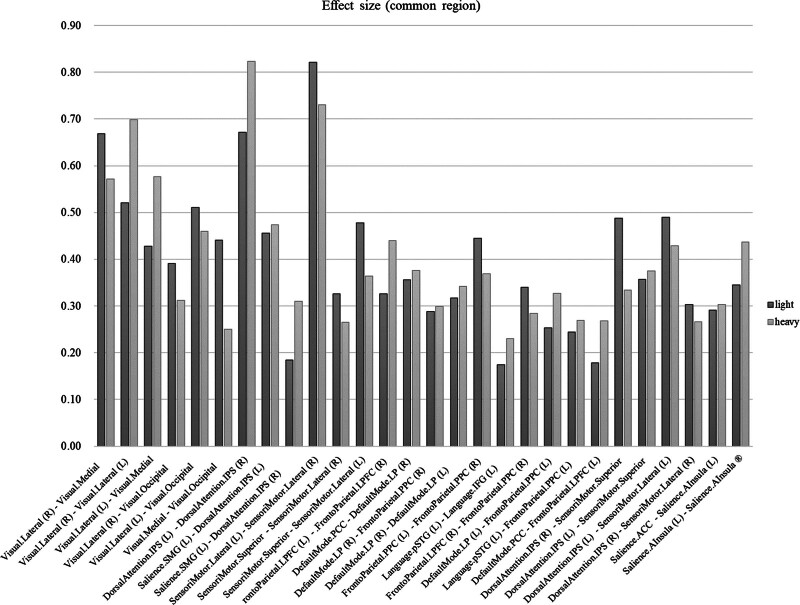
Comparison result graph of the effect size of the network connection, which is a common region connection under both conditions.

Figure 3 shows the comparison result graph of the effect size of the network connection, which is common under both conditions. The connection in which the effect size decreased when flexion and extension of the elbow joint was performed with 1000 g objects rather than 500 g objects was Visual.lateral (right, R)–Visual.medial, Visual.medial–Visual.occipital, SensoriMotor.superior–SensoriMotor.lateral (left, L), FrontoParietal.LPFC (R)–FrontoParital.PCC (R), and DorsalAttention.IPS (R)–SensoriMotor.superior. The connection in which the effect size increased when flexion and extension the elbow joint was performed with 1000 g objects rather than 500 g objects was Visual.lateral (L)–Visual.medial, Salience.SMG (L)–DorsalAttention.IPS (R), Language.pSTG (L)–Language.IFG (L), and Salience.AInsula (L)–Salience.AInsula (R).

The FCs that showed the highest effect size when performing the exercise with 500 g objects were DorsalAttention.FEF (L)–SensoriMotor.superior, Language.IFG (L)–Language.IFG (R), and DorsalAttention.IPS (R)–SensoriMotor.lateral. On the other hand, the FCs that showed the highest effect size when performing elbow exercises with 1000 g objects were Language.pSTG (L)–Language.pSTG (R) and Salience.SMG (R)-DorsalAttention.IPS (R).

## 4. Discussion

In this study, the results of FC between various areas were extracted when flexion and extension of the elbow were repeatedly performed with 500 g and 1000 g weight in hand.

Visual.lateral (R)–Visual.medial, Visual.medical–Visual.occipital, SensoriMotor.superior–SensoriMotor.lateral (L), FrontoParital.LPFC (R)–FrontoParital.PPC (R), DorsalAttention.IPS (R)–SensoriMotor.superior connection reduced the effect size between areas when flexion and extension of the elbow joint with 1000 g objects more than 500 g objects were carried out. The visual network, including lateral, medial, and occipital areas, is primarily responsible for processing visual information. The decrease in correlation might indicate a shift in focus from visual processing to motor execution as the task becomes more challenging with a heavier weight.^[6]^ The brain reallocates resources to areas more directly involved in motor control and proprioception, leading to a relative reduction in the synchronization of visual regions. In the case of the SensoriMotor.superior and SensoriMotor.lateral (L) regions, it plays a role directly involved in controlling and executing movement, and the connection between the 2 regions is part of the sensory motor network.^[7]^ When the task’s difficulty increases (due to the heavier weight), there might be a need for more refined and focused motor control. This could lead to a differentiation in the activity of various parts of the sensorimotor cortex, reflecting a more specialized or localized motor processing strategy. The LPFC and the PPC are key components of the FPN involved in high-level executive functions and motor planning.^[8,9]^ The decreased correlation (FrontoParietal.LPFC [R]–FrontoParietal.PCC [R]) might suggest a shift in the cognitive strategy as the task becomes more complex. It could reflect a more distributed or varied approach to motor planning and execution, where different aspects of the task are processed more independently. DAN and SensoriMotor integration: The DAN, which includes the right IPS, is involved in directing attention, particularly in spatial and movement-related contexts.^[10,11]^ The SensoriMotor areas are responsible for executing and coordinating movement.^[12]^ In tasks involving greater physical demands, such as lifting a heavier weight, there might be a more focused and localized engagement of the SensoriMotor areas. This could potentially lead to a decrease in the correlation with the dorsal attention areas, as the brain allocates more resources to direct motor control rather than broader attentional processes.

The connection in which the effect size increased when flexion and extension of the elbow joint was performed with 1000 g objects rather than 500 g objects was Visual.lateral (L)–Visual.medial, Salience.SMG (L)–DorsalAttention.IPS (R), Language.pSTG (L)–Language.IFG (L), and Salience.AInsula (L)–Salience.AInsula (R). Visual.lateral (L)–Visual.medial connection is part of the visual processing system in the brain. The increased correlation might be due to the heightened visual attention and spatial awareness required when handling heavier weights, as the brain needs to coordinate more complex visual-motor integration.^[13]^ The salience network, involving the SMG, is important for detecting and filtering salient stimuli,^[14,15]^ while the DAN, involving the IPS, is crucial for top–down attentional control.^[16]^ The increased correlation (Salience.SMG [L]–DorsalAttention.IPS [R]) could suggest a greater need for attentional focus and the integration of sensory information when the physical demand is higher. Language.pSTG (L)–Language.IFG (L) connection is involved in language processing, with the pSTG and IFG playing roles in the comprehension and production of language, respectively.^[17]^ The change in correlation could be related to the internal verbalization or self-talk strategies often used in concentration and effort modulation during challenging tasks. The Anterior Insula is part of the salience network and is involved in integrating sensory information with emotional and cognitive processing.^[18]^ An increased correlation (Salience.AInsula [L]–Salience.AInsula [R]) here could indicate a heightened state of bodily awareness and emotional processing due to the increased challenge of lifting heavier weights.

The connection that showed the highest correlation coefficient when performing elbow exercise with a 500-g object was DorsalAttention.FEF (L)–SensoriMotor.superior, Language.IFG (L)–Language.IFG (R), and DorsalAttention.IPS (R)–SensoriMotor.lateral. DorsalAttention.FEF and SensoriMotor.superior: This connection involves areas associated with motor planning and execution. The FEF, part of the DAN, is crucial for saccadic eye movements and attentional focus, while the SensoriMotor area is central to motor control. The high correlation could be indicative of the integrated motor and attentional processes required to maintain grip and posture while holding an object, even as light as 500 g. Language.IFG connection: The IFG is a critical region for language processing. High interhemispheric connectivity (between left and right IFG) suggests a strong coordination between these areas during the task. Although the IFG is primarily known for language, it also plays a role in cognitive processes like working memory, which might be engaged in maintaining the strategy or instructions related to the task. DorsalAttention.IPS and SensoriMotor.lateral: The IPS, part of the DAN, is involved in spatial awareness and attention, while the SensoriMotor area relates to movement control. High connectivity here might reflect the coordination between spatial attention and precise motor control, which is essential when manipulating objects.

On the other hand, when performing elbow exercises with 1000 g of objects, the highest correlation coefficients were Language.pSTG (L)–Language.pSTG (R) and Salience.SMG (R)–DorsalAttention.IPS (R). Language.pSTG (L)–Language.pSTG (R): The pSTG plays a pivotal role in language processing and comprehension, involving both hemispheres of the brain. The left pSTG is traditionally associated with language processing, especially in understanding spoken words.^[19]^ The right pSTG, while less dominant in language, contributes to the processing of complex aspects of language, such as intonation and prosody.^[19]^ The high correlation between the left and right pSTG could suggest a coordinated bilateral engagement during language tasks, possibly more pronounced when manipulating objects (like a 1000-g ball) that might require additional cognitive and sensory integration. Salience.SMG (R)–DorsalAttention.IPS (R): The right SMG is part of the salience network and is involved in attention, social cognition, and empathy.^[20]^ The right IPS, a part of the DAN, plays a crucial role in controlling spatial attention and guiding motor actions. A high correlation (Salience.SMG [R]–DorsalAttention.IPS [R]) between these regions suggests a strong interaction between attentional focus and the processing of sensory and social stimuli. During tasks that require physical manipulation (like holding weights), this correlation might indicate a heightened coordination between attentional processes and sensory motor integration. The observed high correlation in these connections likely reflects the increased demand for integrated cognitive and sensory motor processing during the task of holding and manipulating weights. This demand necessitates a stronger and more coordinated interaction between these brain regions.

Table 2 shows the negative values of *t* score and effect size among the network connections that appeared when elbow bending and stretching were performed with 1000 g objects. Negative correlations in FC are indicative of inverse or anticorrelated relationships between the activities of different brain regions. This means that when one region’s activity increases, the activity in the other region decreases, and vice versa. This kind of functional relationship is essential for the balance and efficiency of brain networks. It allows for a kind of push-and-pull dynamic where different networks can become active or quiet depending on the task requirements.^[21,22]^ In the case of the specific regions you have mentioned, such as the Default Mode network’s LP region, the FPN’s PPC and LPFC, DAN’s FEF, and SensoriMotor network’s lateral areas, negative correlations could reflect a complex interplay where these networks counterbalance each other to optimize performance during the task. For instance, regions involved in attention and sensory processing might become more active, requiring simultaneous downregulation of regions involved in default mode processing, which are more active during rest.^[21–23]^

## 5. Conclusions

This study analyzed the impact of changes in object weight (500 g and 1000 g) on functional brain connectivity during elbow flexion and extension tasks. The results showed that increasing the weight during the task led to changes in the strength of connectivity between specific networks, with some connections weakening while others strengthened. For instance, significant patterns were observed in the connectivity between the left FEF and the superior SensoriMotor area, as well as between the pSTG and the IFG, depending on the weight used. These findings suggest that the brain reallocates resources and adjusts the coordination between SensoriMotor integration and attention-regulation networks based on the demands of the motor task. Specifically, when handling heavier objects, there was an increase in cooperation among visual, attention, and language networks, while basic SensoriMotor connectivity showed a tendency to decrease. This reflects the brain’s adaptive strategies in response to increased task difficulty. This study provides foundational insights into these changes in brain networks, which can inform the design of rehabilitation programs and the optimization of motor skill training. Future research will include a broader range of variables such as age, gender, and physical fitness, as well as an expanded range of object weights, to further explore these findings in greater depth.

## Author contributions

**Conceptualization:** Jin-Seung Choi, Mi-Hyun Choi.

**Investigation:** Jin-Seung Choi, Mi-Hyun Choi.

**Methodology:** Jin-Seung Choi, Mi-Hyun Choi.

**Writing—original draft:** Jin-Seung Choi, Mi-Hyun Choi.

**Data curation:** Mi-Hyun Choi.

**Formal analysis:** Mi-Hyun Choi.

**Funding acquisition:** Mi-Hyun Choi.

**Project administration:** Mi-Hyun Choi.

**Resources:** Mi-Hyun Choi.

**Software:** Mi-Hyun Choi.

**Supervision:** Mi-Hyun Choi.

**Validation:** Mi-Hyun Choi.

**Visualization:** Mi-Hyun Choi.
